# Axillary lymphadenopathy at the time of COVID-19 vaccination: ten recommendations from the European Society of Breast Imaging (EUSOBI)

**DOI:** 10.1186/s13244-021-01062-x

**Published:** 2021-08-20

**Authors:** Simone Schiaffino, Katja Pinker, Veronica Magni, Andrea Cozzi, Alexandra Athanasiou, Pascal A. T. Baltzer, Julia Camps Herrero, Paola Clauser, Eva M. Fallenberg, Gábor Forrai, Michael H. Fuchsjäger, Thomas H. Helbich, Fleur Kilburn-Toppin, Christiane K. Kuhl, Mihai Lesaru, Ritse M. Mann, Pietro Panizza, Federica Pediconi, Ruud M. Pijnappel, Tamar Sella, Isabelle Thomassin-Naggara, Sophia Zackrisson, Fiona J. Gilbert, Francesco Sardanelli

**Affiliations:** 1 Unit of Radiology, IRCCS Policlinico San Donato, San Donato Milanese, Italy; 2 Department of Biomedical Imaging and Image-guided Therapy, Division of General and Pediatric Radiology, Research Group: Molecular and Gender Imaging, Medical University of Vienna, Wien, Austria; 3 Department of Radiology, Breast Imaging Service, Memorial Sloan Kettering Cancer Center, New York, NY USA; 4 Department of Biomedical Sciences for Health, Università degli Studi di Milano, Milan, Italy; 5 Breast Imaging Department, MITERA Hospital, Athens, Greece; 6 Área de Salud de la Mama , Ribera Salud Grupo, Valencia, Spain; 7 Department of Diagnostic and Interventional Radiology, School of Medicine & Klinikum Rechts der Isar, Technical University of Munich (TUM) , München , Germany; 8 Department of Radiology, Duna Medical Center, Budapest, Hungary; 9 Division of General Radiology, Department of Radiology, Medical University Graz, Graz, Austria; 10 Department of Radiology, University of Cambridge, Cambridge, UK; 11 University Hospital of Aachen, Rheinisch-Westfälische Technische Hochschule Aachen, Aachen, Germany; 12 Radiology and Imaging Laboratory, Fundeni Institute, Bucharest, Romania; 13 Department of Medical Imaging, Radboud University Nijmegen Medical Centre, Nijmegen, The Netherlands; 14 Department of Radiology, The Netherlands Cancer Institute, Amsterdam, The Netherlands; 15 Breast Imaging Unit, IRCCS Ospedale San Raffaele, Milan, Italy; 16 Department of Radiological, Oncological, and Pathological Sciences , Università degli Studi di Roma “La Sapienza” , Rome, Italy; 17 Department of Imaging, University Medical Centre Utrecht, Utrecht University, Utrecht, The Netherlands; 18 Department of Diagnostic Imaging, Hadassah Hebrew University Medical Center, Jerusalem, Israel; 19 Department of Radiology, Hôpital Tenon APHP, Sorbonne Université, Paris, France; 20 Diagnostic Radiology, Department of Translational Medicine, Skåne University Hospital, Lund University, Malmö, Sweden

**Keywords:** COVID-19 vaccines, Lymphadenopathy, Mammography, Ultrasonography (breast), Magnetic resonance imaging

## Abstract

Unilateral axillary lymphadenopathy is a frequent mild side effect of COVID-19 vaccination. European Society of Breast Imaging (EUSOBI) proposes ten recommendations to standardise its management and reduce unnecessary additional imaging and invasive procedures: (1) in patients with previous history of breast cancer, vaccination should be performed in the contralateral arm or in the thigh; (2) collect vaccination data for all patients referred to breast imaging services, including patients undergoing breast cancer staging and follow-up imaging examinations; (3) perform breast imaging examinations preferentially before vaccination or at least 12 weeks after the last vaccine dose; (4) in patients with newly diagnosed breast cancer, apply standard imaging protocols regardless of vaccination status; (5) in any case of symptomatic or imaging-detected axillary lymphadenopathy before vaccination or at least 12 weeks after, examine with appropriate imaging the contralateral axilla and both breasts to exclude malignancy; (6) in case of axillary lymphadenopathy contralateral to the vaccination side, perform standard work-up; (7) in patients without breast cancer history and no suspicious breast imaging findings, lymphadenopathy only ipsilateral to the vaccination side within 12 weeks after vaccination can be considered benign or probably-benign, depending on clinical context; (8) in patients without breast cancer history, post-vaccination lymphadenopathy coupled with suspicious breast finding requires standard work-up, including biopsy when appropriate; (9) in patients with breast cancer history, interpret and manage post-vaccination lymphadenopathy considering the timeframe from vaccination and overall nodal metastatic risk; (10) complex or unclear cases should be managed by the multidisciplinary team.

## Key points

- Worldwide COVID-19 vaccination campaigns are currently underway and could become commonplace.
- Post-vaccination COVID-19 lymphadenopathy has been reported in up to 16% of cases.
- Breast imaging should be performed before or 12 weeks after the last dose of vaccine.
- European Society of Breast Imaging (EUSOBI) provides ten recommendations to manage COVID-19 post-vaccination unilateral axillary lymphadenopathy.

## Background

Alongside staple preventive measures such as physical distancing, consistent use of face masks, prompt deployment of testing, tracing and isolation protocols [1], a quick and effective rollout of vaccination campaigns throughout the world represents the key element to contain the SARS-CoV-2 pandemic and begin a transition towards normal social and economic activity [2–4]. As of July 27, 2021, the World Health Organization lists 292 candidate vaccines in clinical development, 184 in the pre-clinical phase and 108 in the clinical phase [5]. The European Commission, through the European Medicines Agency, has up to now issued four conditional marketing authorisations for vaccines developed by Pfizer–BioNTech, Moderna, AstraZeneca, and Janssen Pharmaceuticals [6, 7]. These companies used different development strategies, including messenger RNA (mRNA)-based and adenovirus vector-based vaccines, and proposed different vaccination programs (Table 1). Despite the unquestionable positive protective effect, a number of widespread local and systemic reactions, mostly mild and following the second dose, have been observed both in clinical trials and in the population-wide rollout of vaccines, the main being pain at the site of injection, ipsilateral axillary lymph node enlargement, tiredness, headache, and fever [7–9]. In this paper, we focus on the reaction which could have a sizable impact on breast imaging, namely axillary lymph node enlargement, and provide recommendations on the management of this side effect.

**Table 1:** Main characteristics of the four COVID-19 vaccines approved in Europe as of July 28, 2021

Developer	Commercial name	Type	Number of doses	Dosage interval
Pfizer–BioNTech	Comirnaty	mRNA-based	2	3–12 weeks
Moderna	COVID-19 Vaccine Moderna	mRNA-based	2	4 weeks
AstraZeneca	Vaxzevria	Adenovirus vector-based	2	4–12 weeks
Johnson and Johnson - Janssen Pharmaceuticals	COVID-19 Vaccine Janssen	Adenovirus vector-based	1	–

## The pre-COVID-19 scenario

In breast imaging, axillary lymphadenopathy can be detected at mammography, digital breast tomosynthesis, ultrasonography, magnetic resonance imaging (MRI), and chest computed tomography or positron emission tomography/computed tomography staging exams, being potentially related to a wide spectrum of benign (e.g., mastitis, breast abscess, infected skin lesions, cat-scratch fever) and malignant (e.g., breast cancer, lymphoma, melanoma, ovarian cancer) conditions [10, 11]. According to the 2013 edition of the American College of Radiology Breast Imaging Reporting and Data System (ACR BI-RADS) Atlas [11], isolated unilateral axillary lymphadenopathy without underlying abnormal breast findings or known infection or inflammation should be considered suspicious (BI-RADS 4 category). In this scenario, an additional imaging work-up to rule out breast cancer is recommended and, if negative, fine-needle aspiration or core biopsy of the enlarged lymph node should be performed. Of note, occult primary breast cancer presenting with lymph node metastases but negative conventional imaging accounts for up to 1% of all breast cancers [12, 13], although in 75–85% of cases the cancer is detectable on breast MRI [14, 15].

## The post-COVID-19 scenario and the relationship with vaccination

Just as lymphadenopathy is a common feature of severe COVID-19 presentation [16], lymph node swelling is a common side effect of vaccinations that evoke a robust immune response [17, 18] and has been described after COVID-19 vaccination in the axilla ipsilateral to the injected deltoid muscle [19]. In the phase III trial of the Moderna vaccine [20], palpable axillary lymphadenopathy ipsilateral to the vaccination arm was reported in 11.6% of recipients after the first dose and in 16.0% of recipients after the second dose, occurring within 2–4 days and with a median duration of 1–2 days. Conversely, in the phase III trial of the Pfizer–BioNTech vaccine [21], lymphadenopathy was reported in 0.3% of recipients, occurred within 2–4 days, and lasted approximately 10 days. Notably, both these trials reported only clinically assessed lymphadenopathy and probably underestimated the rate of subclinical lymphadenopathy, which could be detected by imaging [22]. Of note, a recent study on 169 positron emission tomography–computed tomography examinations has revealed that as many as 29% of examined patients still showed avid uptake of fluorodeoxyglucose in ipsilateral lymph nodes 7–10 weeks after the second dose of the Pfizer–BioNTech vaccine [23]. Since the beginning of the vaccination campaigns in the USA and Europe, several case series have described lymphadenopathy presenting both as a palpable mass or as an incidental finding during routine breast imaging after COVID-19 vaccination [24–27].

**Fig. 1:**
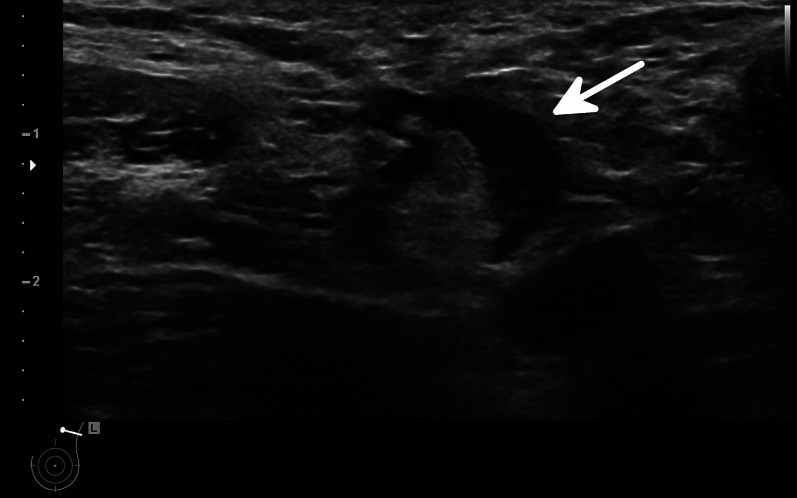
Ultrasonography of the left axilla showing an enlarged 17 mm reactive lymph node in a 45-year-old woman about a week after receiving the first dose of the Vaxzevria COVID-19 vaccine. Note the asymmetrical cortical thickening (white arrow) associated with a well-represented central fatty hilum

**Fig. 2:**
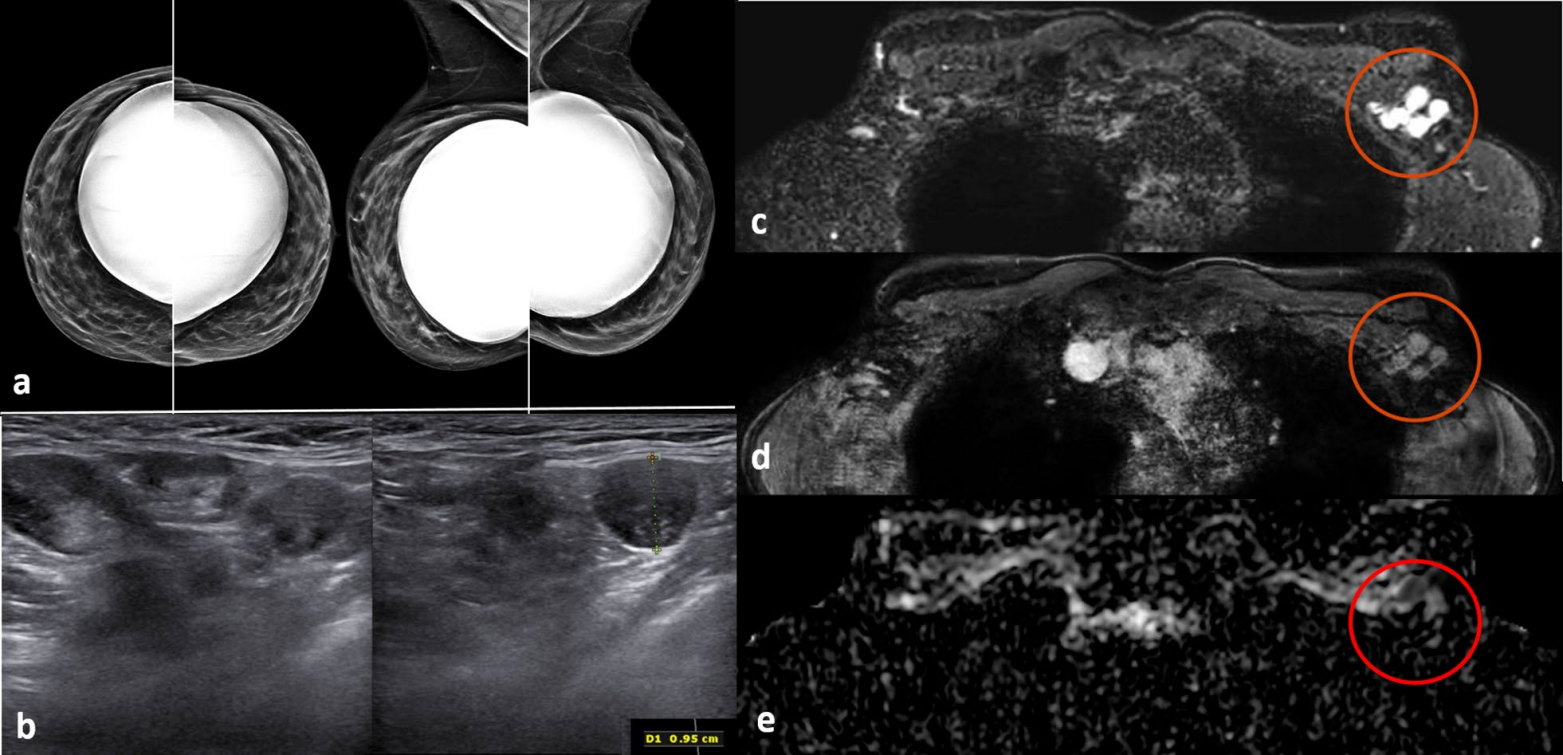
Screening mammography performed in a 44-year-old woman with a positive family history for breast cancer (mother and aunt), bearing implants for aesthetic purposes. Mammography (**a**) was considered negative. Breast ultrasonography was also performed because of her family history and high breast density (ACR category *d*). While ultrasonography was negative for both breasts, multiple round, enlarged, hypoechoic lymph nodes (measuring up to 1 cm in axial diameter), with a thickened (< 3 mm) cortex, were seen in the left axilla (**b**). There were no skin changes and there was no history of any infection or trauma. On the right side, axillary lymph nodes were normal. Because of her family history and the presence of breast implants, magnetic resonance imaging was performed (**c** T2-weighted short-time inversion recovery; **d** fat-sat contrast-enhanced T1-weighted gradient-echo; **e** apparent diffusion coefficient map). No suspicious mass or non-mass lesions were seen in both breasts. Implants showed no signs of rupture (not shown). In the left axilla, multiple enlarged lymph nodes were well visible in **c** and **d** (red circles); on the apparent diffusion coefficient map (**e**, red circle), they mainly exhibited low signal (restricted diffusivity). When an ultrasound-guided biopsy of the most suspicious lymph node was proposed, the patient mentioned that she had a Comirnaty COVID-19 vaccination one week before in the left arm. The attending radiologist was more than surprised to hear this, as at that time, a COVID-19 vaccination was only administered to people older than 70 years. Follow-up performed four weeks after the second vaccination was negative and showed no residual enlarged lymph nodes

Thus, when facing an incidental or symptomatic unilateral axillary lymphadenopathy, while malignancy remains the most critical aetiology, COVID-19 vaccination needs to be recognised as a potential differential diagnosis. Examples of axillary lymphadenopathy after COVID-19 vaccination are presented in Figs. 1 and 2.

## Recommendations

Since worldwide mass vaccination campaigns against COVID-19 are currently underway, breast radiologists should be aware of reactive axillary lymphadenopathy as a possible side effect of vaccination, to limit patients’ anxiety and avoid unnecessary diagnostic imaging and invasive procedures. We carefully considered the Recommendations for the Management of Axillary Adenopathy in Patients with Recent COVID-19 Vaccination [28] issued by the Society of Breast Imaging, the statements by Becker et al. [22], Edmonds et al. [29], and Lehman et al. [30, 31].

EUSOBI provides the following recommendations regarding general issues (number 1, 2), asymptomatic subjects, including women attending screening programs (number 3), cases with symptoms or imaging-detected findings (number 4–9), and complex cases (number 10).

1. In patients with previous history of breast cancer, vaccine injection (both doses for two-doses vaccines) should be performed in the contralateral arm or in the anterolateral thigh.
2. COVID-19 vaccination data (vaccination status, date, dose, injection site) of all patients presenting for breast imaging with any modality should be collected and made available to radiologists, including the cases of breast imaging performed for cancer staging and of follow-up imaging examinations.
3. Breast examinations should be preferentially performed before the first dose of a COVID-19 vaccine or at least 12 weeks after the injection. For vaccines with a two-dose schedule, the 12-weeks rule applies from the day of the second injection.
4. In patients newly diagnosed with breast cancer, all necessary breast imaging examinations with any modality must be performed without any delay due to vaccination, taking into consideration the risk of false positive lymph node findings.
5. The contralateral axilla and both breasts should be clinically examined using appropriate imaging to exclude malignancy in all patients with axillary symptoms and in all cases of imaging-detected unilateral axillary lymphadenopathy before vaccination or at least 12 weeks after.
6. In patients with or without previous breast cancer history, imaging-detected suspicious axillary lymphadenopathy contralateral to the vaccination side should be managed according to standard work-up protocols, including, when necessary, tissue sampling.
7. In patients without breast cancer history and no suspicious breast imaging findings, imaging-detected unilateral axillary lymphadenopathy on the same side of recent COVID-19 vaccination (i.e., within 12 weeks) should be managed according to the clinical setting (Table 2). In asymptomatic patients it should be classified as a benign finding (BI-RADS 2) and no further work-up should be pursued. In case of patients reporting symptoms of axillary lymphadenopathy more than 12 weeks after vaccination, ultrasound examination of the axilla is recommended. In patients with axillary symptoms, incidental unilateral axillary lymphadenopathy ipsilateral to the vaccination side without any suspicious finding in the breast should be classified as a probably benign finding (BI-RADS 3), requiring a 12-week follow-up. In case of persistent suspicion at this 12-week follow-up, ACR BI-RADS recommendations for the management of axillary lymphadenopathy should be followed, with further work-up including, when necessary, tissue sampling [11].
8. In patients without breast cancer history, incidental unilateral axillary lymphadenopathy after COVID-19 vaccination coupled with ipsilateral suspicious findings in the breast at any imaging modality should be managed according to clinical practice, including biopsy when appropriate [11].
9. In patients with personal breast cancer history, lymphadenopathy after vaccination should be interpreted considering the time since vaccination and overall nodal metastatic risk (cancer type, location, stage, etc.) [32]. For patients at low risk of axillary or supraclavicular nodal metastases in whom the lymphadenopathy is overwhelmingly more likely due to the vaccination than to the underlying neoplasm (considering time frame, pain, type, and location of cancer), a cautious management strategy without default follow-up imaging is appropriate. Short-interval follow-up imaging with ultrasonography (with at least a 12-week delay) may be performed in patients with higher risk of metastatic lymphadenopathy (e.g., breast cancer, head and neck cancer, upper extremity/trunk melanoma or lymphoma). Node biopsy should be considered in the setting of high nodal metastatic risk when immediate histopathologic confirmation is necessary for timely patient management.
10. All complex or unclear cases (e.g., axillary lymphadenopathy ipsilateral to the cancer and the side of vaccination within 12 weeks after vaccination in patients with previous bilateral breast cancer; vaccinations performed on different sides) should follow a personalised management, considering the risk of malignant lymphadenopathy, opting for tissue sampling when appropriate after multidisciplinary team discussion.

**Table 2:** Management of incidental unilateral axillary lymphadenopathy after recent (within 12 weeks) COVID-19 vaccination

Patients	Clinical context	Management
Without any history of breast cancer	No symptoms No suspicious breast findings at imaging	Unilateral axillary lymphadenopathy ipsilateral to the vaccination side should be classified as a benign (BI-RADS 2) finding and no further work-up should be pursued. Ultrasonography should be performed in case of symptoms of axillary lymphadenopathy more than 12 weeks after vaccination
	Breast imaging for breast symptoms No suspicious breast findings at imaging	Unilateral axillary lymphadenopathy ipsilateral to the vaccination side should be classified as a probably benign (BI-RADS 3) finding, and clinical follow-up of the axilla is indicated. In case of symptoms of axillary lymphadenopathy more than 12 weeks after vaccination, ACR BI-RADS recommendations should be followed for the management of axillary lymphadenopathy
With personal breast cancer history	Any context	Avoid vaccination at the breast cancer side. Manage unilateral axillary lymphadenopathy ipsilateral to the vaccination side according to overall nodal metastatic risk. For patients at low risk, define a case-by-case cautious management strategy. For patients at high risk, perform short-interval follow-up imaging with ultrasonography with at least a 12-week delay post vaccination, with node biopsy when necessary

*BI-RADS* Breast Imaging Reporting and Data System, *ACR* American College of Radiology

Recommendation number 3 could be difficult to be applied in organised population-based screening programs. In this case, we suggest to carefully apply recommendation number 2.

These recommendations should be applied to both female and male patients. The latter, of course, do not undergo breast cancer screening with the exception of *BRCA2* mutation carriers, who could be included in high-risk screening programs [33].

Of note, sites for vaccination alternative to the proximal arm (such as the thigh) [31] could be considered to avoid most breast care-related problems ensuing from the current vaccine administration practice.

## Conclusions

Since the rollout of COVID-19 vaccines is rapidly proceeding, radiologists will increasingly encounter in their practice COVID-19 vaccination-induced lymphadenopathy detected by breast imaging [19]. Moreover, the emergence of new SARS-CoV-2 variants and the unclear durability of vaccine-induced immunity [34] will likely lead to re-vaccination or to the administration of new vaccines, further extending this issue. Thus, further research and adherence to evidence-based recommendations are paramount to standardise the management of these findings, avoiding unnecessary additional imaging and invasive procedures.

## Data Availability

Not applicable.

## Abbreviations

ACR: American College of Radiology
BI-RADS: Breast Imaging Reporting and Data System
COVID-19: Coronavirus disease 2019
EUSOBI: European Society of Breast Imaging
MRI: Magnetic resonance imaging
SARS-CoV-2: Severe acute respiratory syndrome coronavirus 2
